# Efficient Electrochemical Reforming of Water-Insoluble C‑Only Plastic Wastes

**DOI:** 10.1021/acssuschemeng.5c00907

**Published:** 2025-05-27

**Authors:** Tayebeh Esmaeili, Julian Hörndl, Simone Pokrant, Theresa Bartschmid, Amin Farhadi, Gilles R. Bourret

**Affiliations:** Department of Chemistry and Physics of Materials, 27257 University of Salzburg, Jakob Haringerstraße 2a, A-5020 Salzburg, Austria

**Keywords:** reforming, hydrogen, plastic waste, polystyrene, polypropylene, electrocatalysis

## Abstract

We report here the efficient electrochemical reforming of hydrocarbon polymer wastes, i.e. composed of C–C and C–H bonds only, in aqueous solution at 3 V. Anodic degradation of these chemically resilient wastes is achieved with Faradaic efficiencies of up to 32% on a Ni/Sb-doped SnO_2_ electrode. The hydrophobic plastic particles, initially present as large aggregates, are solubilized during the early stages of the reaction, which is essential to achieve high reforming efficiencies. Cathodic H_2_ generation is demonstrated with Faradaic and energy efficiencies of up to 57% and 30%, respectively. Under optimized conditions, electroreforming requires ca. 0.10 kWh/g of plastic degraded, which is >120 times more efficient than that previously reported on boron-doped diamond anodes. If scaled up, energy costs as low as ca. 2000$/ton could be achieved, while the H_2_ generated could cover up to ca. 70% of these costs. CO_2_ emissions, expected to be ranging from 1.65 to 13.02 kg_CO2eq_/kg_H2_, are competitive with conventional plastic-to-H_2_ high-temperature processes. Our results support the industrial potential of plastic electroreforming to efficiently treat chemically resilient plastic wastes.

## Graphical abstract

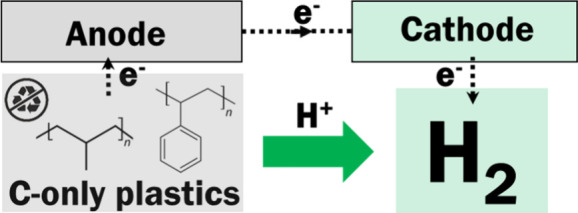

## 1 Introduction

Each year, more than 400 million tons of plastics are produced from fossil fuels.^1,2^ Designed and engineered to resist heat, chemicals and light, plastics are not disposed in a sustainable manner, and largely end up in landfills, incinerated, or littered in the environment.^1^ Only about 10% is recycled into materials of lower quality and value that will ultimately share the same fate.^2^ Overall, the mismanagement of plastic waste has resulted in the widespread contamination of micro- and nanoplastics across the globe, and contributed to climate change due to the emission of greenhouse gases during plastic incineration and their slow decomposition.^3–5^ By recovering the energy released during plastic combustion, waste-to-energy incinerators can mitigate CO_2_ emission and limit the micro/nanoplastic pollution that results from landfilling.^6^ However, the process is not CO_2_ neutral, does not generate high-value chemicals such as H_2_, can still emit small particles and toxic chemicals causing adverse health effects, and generates ashes that must be properly disposed.^7–9^

Instead, upcycling offers a promising alternative to convert plastic waste into high-value chemicals, but often requires high-temperature and/or multistep processes that are energy demanding.^6,10–18^ The “soft” conversion of C-only plastics, solely composed of C and H atoms, has been particularly difficult because of their high chemical stability.^11,19^ Enzymatic degradation of plastics has been proposed as softer and simpler approach. It is a slow process that requires several days or weeks, it cannot completely degrade C-only plastics, and has not been used to produce hydrogen from such wastes.^20,21^ Photocatalysis has shown some success in breaking down these resilient plastics,^22–25^ which make up over 60% of all plastic wastes.^23^ However, technical issues, such as limited light absorption, catalyst degradation, and challenges in gas separation, have hindered the scalability of this approach. Instead, electrocatalytic systems powered by renewable energy have emerged as a more practical approach, allowing for easier separation of gas products, while being amenable to the installation of large scale plants.^26–28^ For instance, advancements in water electrolysis technology has made it economically competitive with steam methane reforming, once carbon capture and storage are taken into account.^28^ For now, conventional water electrolyzers spend a considerable amount of energy to generate O_2_ at the anode side, which has no financial value, while producing H_2_ with an energy efficiency (EE) of ca. 60% due to the overpotential necessary to drive the O_2_ evolution reaction.^28^ This energy could instead be redirected to break down plastics at room temperature. Although electrocatalysis has been used to convert a hydrolyzable oxygen-containing polymer, e.g. polyethylene terephthalate (PET), into H_2_ and potassium diformate,^29^ there are significant challenges in applying this technique to ″C-only″ plastics, which are immune to hydrolysis, are water-insoluble, and lack reactive bonds that can be broken easily.

Recent research attempted to use boron-doped diamond (BDD) electrodes to degrade synthetic water-soluble polystyrene (PS) particles in water, achieving high degradation but at an unsustainable energy cost.^30,31^ Kiendrebeogo et al. report the electromineralization of PS microspheres at a concentration of 0.1 g/L, with estimated energy costs of over $250,000 per ton of treated plastic (see Supporting Information Note S1).^30^ Such high costs arise from the extreme voltage used, e.g. > 11 V, and the low mineralization Faradaic efficiency achieved: FE_PS‑to‑CO2_ ∼ 1%.^30^ More recently, Pérez-López et al. studied the degradation of water-soluble 150 nm PS nanoparticles at a concentration of 0.02 – 0.1 g/L with estimated energy costs of over $472,297 to achieve a 91% degradation efficiency (see Supporting Information Note S1 and Table S1).^31^ Both approaches required prohibitively high energy consumption, and only investigated water-soluble synthetic polystyrene particles that do not accurately represent typical, hydrophobic plastic wastes, limiting their practical applications to treat solid plastic wastes.

Herein, we report a tandem plastic reforming electrolyzer combining plastic waste mineralization at a Ni/Sb-doped SnO_2_ (NATO) anode with the cathodic generation of H_2_ at a Pt cathode. This *electrochemical reforming* (Figure 1) works at room temperature, in an aqueous electrolyte, and can convert chemically resilient C-only plastic wastes into valuable H_2_ (ca. 1900$/ton).^32^ NATO anodes were chosen as a cost-effective alternative to BDD electrodes, which have production costs >7000$/m^2^,^33^ because of their low-cost of fabrication and high efficiency at generating reactive HO^•^ radicals (2–5% *FE*_*HO^•^*_, which is >100 times higher than on planar BDD electrodes) that are able to break C–C bonds, and O_3_ (9–14% *FE*_*O3*_).^34–37^

**Figure 1:**
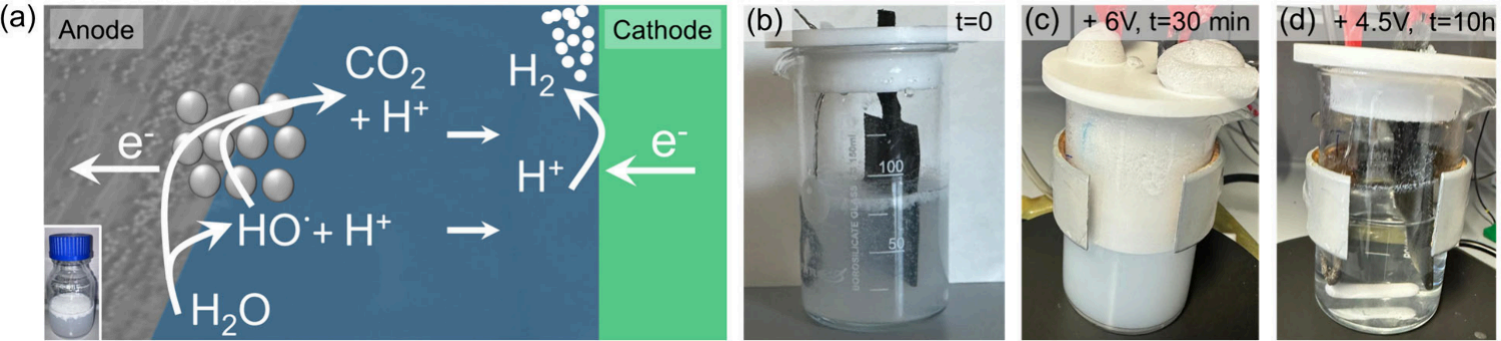
Electroreforming of C-only plastic wastes. (a) Schematic showing coupled anodic mineralization of plastic wastes via direct oxidation and mediated by HO^•^ radicals, and cathodic H_2_ generation. Bottom left inset: Photograph of an aqueous plastic waste stock solution showing significant aggregation of the plastic particles in solution and at the air–water interface. (b-d) Typical photographs of the electrochemical cell filled with an aqueous 0.75 M LiClO_4_ electrolyte containing PS nanoparticles at a concentration of 0.46 g/L: (b) Before electroreforming; (c) After 30 min of electroreforming at 6 V and moderate stirring: Significant foaming occurs, which prevents reliable and efficient electroreforming of the plastic wastes; (d) After 10 h of electroreforming at 4.5 V under vigorous stirring: The plastic electrolyte solution is clear due to efficient mineralization of the plastic particles.

## 2 Materials and Methods

### 2.1 Materials

Lithium perchlorate (99.99%) and antimony­(III) chloride (99%) were purchased from Merck, tin­(IV) chloride pentahydrate(98%), dichloromethane were obtained acquired from Sigma-Aldrich, isopropanol (>98%) from VWR, oxalic acid (98%) from Thermo Scientific, nickel­(II) chloride hexahydrate (98%) from ThermoFisher. All chemicals were used as received. The 60 Ti mesh woven (0.2 mm wire diameter) was purchased from Thermo Scientific.

### 2.2 PS_1_ and PS_2_ Plastic Particle Preparation via Dissolution and Precipitation

Two polystyrene wastes were used: PS_1_, obtained from tape casing (3M), and PS_2_ obtained from Petri dishes (VWR). 160 mg of plastic was dissolved in 16 mL dichloromethane and added to 280 mL isopropanol under vigorous stirring to induce precipitation, thus forming the plastic nanoparticles. After centrifugation at 2000 rpm for 2 min twice, solvent removal and vacuum drying overnight, a white plastic particle powder is obtained. The plastic particle solution was prepared by adding 150 mg of dried plastic powder to 160 mL of Milli-Q water. 960 μL of a 25 μL/10 mL aqueous solution of Triton-X, was then added to the plastic particle stock solution. Ultrasonication was then performed to improve the particle dispersion in the aqueous solution using an ultrasonication finger (from the Hielscher model UP 200 St) with power 10–15 W and 2 min. The PS_1_ and PS_2_ nanoparticle stock solutions were composed of strongly agglomerated nanoparticles with average diameters of 120 ± 40 nm and 105 ± 40 nm, respectively. The particle size distribution was obtained after measuring ca. 100 particles using ImageJ and SEM images. The plastic particle/electrolyte solution was prepared fresh before each electroforming experiment by mixing 40 mL of the stock plastic particle solution with 40 mL of an 1.5 M LiClO_4_ aqueous stock solution. The electroforming solution had a molar concentration of 0.75 M/L LiClO_4_, a concentration of plastic particle of ca. 0.460g/L, and of ca. 0.004 g/L of Triton-X: The amount of Triton-X represents less than 1% of the TOC.

### 2.3 Plastic Particle Powder Preparation via Mechanical Grinding

PS_1_, PS_2_ and PP wastes were carefully ground on a silicon carbide sandpaper (waterproof,320/P400 grit). 35 mg of the resulting powder was then dispersed in 40 mL of Milli-Q water and 40 mL of a 1.5 M LiClO_4_ aqueous stock solution, to obtain 80 mL of the electroforming solution, and used as is. No sonication was used, and Triton-X was not added to this type of plastic particle solutions.

### 2.4 Ni/Sb-Doped SnO_2_ (NATO) Anode Preparation

The titanium grid was etched in a 0.1 M oxalic acid solution at 65 °C for 30 min. After rinsing with deionized water, it was dried in an oven at 80 °C for 30 min. The Ti grid was prepared similarly to previous reports:^36^ It was immersed in the metal precursor solution containing SnCl_2_·5H_2_O (0.5556 M), NiCl_2_·6H_2_O (0.0039 M), and SbCl_3_ (0.015 M). The electrode was left in air for a few minutes, after which the Ti grid was annealed in air in a furnace at 500 °C for 10 min. These steps were repeated a total of five times, after which the grid was annealed in the oven for 1 h at 500 °C. After cooling, the NATO coated Ti grid was ready for use. Energy dispersive X-ray spectroscopy (EDS) confirms doping of the SnO_2_ coating (Figure S1), with an average relative atomic concentration of Ni: A_Ni_=*X*_*Ni*_/*X*_*Sn*_= 3%, where *X*_*Ni*_ and *X*_*Sn*_ are the atomic contents of Ni and Sn, respectively, obtained from EDS measurements. Similarly, we found an average relative atomic concentration of Sb: A_Sb_ = *X*_*Sb*_/*X*_*Sn*_ = 6%. Additionally, both powder X-ray diffraction (XRD) and Raman spectroscopy show the presence of the rutile SnO_2_ phase (Figure S2 and S3).

### 2.5 Electrochemical Experiments

Electrochemical experiments were performed in a 200 mL electrochemical cell with a two-electrode configuration. A Pt mesh was used as the counter electrode and a NATO-coated Ti grid was used as the anode, with an immersed geometrical surface area of ca. 12 cm^2^. The distance between the anode and the cathode was fixed at ca. 1 cm. The cell was filled with 80 mL of plastic electrolyte solution with a constant concentration of 0.75 M LiClO_4_. Electroreforming experiments were performed using a computer-controlled Autolab PGSTAT302N potentiostat. A strong stirring of ca. 1400 rpm was used for most experiments as described in the main text. The charge used during a given electroreforming experiment was obtained from the Autolab software. Each NATO/Ti anode was used for ca. 150 h before being discarded.

### 2.6 Raman Measurements

Raman spectra were collected with a dispersive Raman microscope Thermo DXR2 (Thermo, USA) with a confocal microscope (BX41 by Olympus Corp., Japan). Depolarized laser light with an excitation wavelength of 785 nm was used to record the Raman spectra. The measurement conditions were set to laser powers betwteen 1 mW and 30 mW and a 10x objective, producing a laser spot of 3.1 μm in diameter. Further, spectra were recorded with a 50 μm pinhole, providing a spectral resolution of 4.7–8.7 cm^–1^ across the investigated spectral range between 200 cm^–1^ and 3200 cm^–1^. An exposure time of 5 s and 5 accumulations per spectrum were used during data collection. The samples (solid and powders) were placed on a glass slide covered with aluminum foil to avoid a signal contribution of SiO_2_.

### 2.7 Scanning Electron Microscopy (SEM) and Energy Dispersive X-ray Spectroscopy (EDS)

SEM images were acquired using a Zeiss Ultra Plus 55 equipped with an InLens secondary electron detector. EDS elemental analysis was performed using a 50 mm^2^ silicon drift EDS detector from Oxford Instruments.

### 2.8 X-ray Diffraction (XRD)

XRD analysis was performed at room temperature using a Bruker AXS D8Advance diffractometer with a DaVinci design, featuring a 280 mm goniometer radius, a fast-solid-state Lynxeye detector, and an automatic sample changer. Diffraction patterns were collected with Cu Kα1,2 radiation (λ = 154 pm) radiation (λ=154 pm) over a 2θ range of 5° to 95°, with a step size of 0.02°. Divergence and antiscatter slits were set to 0.3° and 4°, respectively. Phase identification was carried out using Bruker DIFFRAC.EVA V2.1 software.

### 2.9 Quantification of the Polymeric Solid Present at the End of the Electroreforming Experiment

At the end of the electroreforming experiment, the plastic electrolyte solution was filtered on a porous membrane. The membrane was then dried under vacuum for 2 days and weighed to obtain the mass of residual plastic contained in the solution after electroreforming. Control experiments using a known quantity of plastic nanoparticle show that the approach is reliable and gives reproducible weights. The contribution of LiClO_4_ salt content present in the membrane after filtration was negligeable and was thus not taken into account in our calculations.

### 2.10 Total Organic Carbon (TOC) Analysis

TOC analysis was performed at the hydrological laboratory of Salzburg Hydrologische Untersuchungsstelle Salzburg (HUS), Ingenieurbüro für Kulturtechnik and Wasserwirtschaft Laboranalytische Dienstleistungen, a private company expert in the chemical analysis of water samples. The TOC analysis was performed using a TOC-L Series from Shimadzu, which runs on carbon-free synthetic air. The TOC values provided were measured as nonpurgeable organic carbon (NPOC) according to EN 1484, i.e. the acidified samples were purged to remove any dissolved CO_2_ and/or carbonates and injected into a catalytic oven at a temperature >700 °C. The resulting combustion products were analyzed for CO_2_ by a nondispersive infrared (NDIR) gas analyzer. To protect the detector-unit, the analyzer is equipped with a copper-mesh halogen-scrubber that removes any chlorine from the combustion gases. The accuracy of the TOC measurements in the 1–200 mg/L range was verified using standards containing a known amount of potassium hydrogen phthalate that were measured in a single blind study, e.g. the standard concentration was not known by the scientist running the TOC measurements. The TOC of pure Milli-Q water and pure 0.75 M LiClO4 electrolyte solution was below the detection limit, which was 1 mg/L for these measurements.

Additionally, after filtration, the TOC of the pristine, unreacted PS_1_ nanoparticle stock solution is 11.3 mg/L, which shows that (i) the residual organic carbon present as soluble content in the stock solution before electroreforming is small, and (ii) the membrane effectively filters out the plastic nanoparticles.

### 2.11 Calculation of the Plastic Mineralization Faradaic Efficiencies

Faradaic efficiency *FE*_plastic‑to‑CO2_ was determined using eq 1

1 $${FE}_{\mathrm{plastic‐to‐CO2}}=\frac{z_{\mathrm{monomer}}\cdot n_{\mathrm{monomer}}\cdot F}{Q}$$

where *z*_*monoXmer*_ is the number of electrons required to mineralize one monomer, *n*_monomer_ is the number of moles of monomer mineralized, *F* is Faraday’s constant, and *Q* is the total charge passed during the electroreforming experiment.

*z*_monomer_ was estimated assuming the following mineralization reactions for polystyrene (eq 2) and polypropylene (eq 3), where *z*_PS_ = 40 electrons are required to convert one PS monomer, i.e. C_8_H_8_, into CO_2_ and *z*_*PP*_ = 18 electrons are required to convert one PP monomer, i.e. C_3_H_6_, into CO_2_.

2 $$\mathrm{PS:}\;nC_{8}H_{8}+16nH_{2}O\to 8n{\mathrm{CO}}_{2}+40nH^{+}+40ne^{-}$$

3 $$\mathrm{PP:}\;nC_{3}H_{6}+6nH_{2}O\to 3n{\mathrm{CO}}_{2}+18nH^{+}+18ne^{-}$$

The reforming reaction of PS and PP can thus be estimated with eqs 4 and 5, respectively:

4 $$\mathrm{PS:}\;nC_{8}H_{8}+16nH_{2}O\to 8n{\mathrm{CO}}_{2}+20nH_{2}$$

5 $$\mathrm{PP:}\;nC_{3}H_{6}+6nH_{2}O\to 3n{\mathrm{CO}}_{2}+9nH_{2}$$

Additionally, *n*_monomer_, e.g. the number of monomer mineralized, was calculated according to eq 6

6 $$n_{\mathrm{monomer}}=m_{\mathrm{plastic mineralized}}\times M_{W}\left(\mathrm{monomer}\right)$$

where *M*_*W*_(monomer) is the molecular weight of the monomer, e.g. *M*_*w*_(PS monomer = C_8_H_8_) = 104.1 g mol^–1^, and *m*_plastic mineralized_, the mass of plastic mineralized, is calculated according to eq 7

7 $$m_{\mathrm{plastic mineralized}}=m_{i}-m_{f}-m_{TOC}$$

where *m*_*i*_ is the initial mass of polymer present in the electrolyte, *m*_*f*_ the residual mass after electoreforming, calculated by weighing the residual solid content, and *m*_TOC_ the mass of residual polymer and organics present in solution calculated using the TOC content present in the electrolyte after filtration. *m*_TOC_ was estimated by assuming that the carbon was present chemically as the pristine plastic, i.e. a TOC of 10 mg/L corresponds to 0.8 mg of carbon in the 80 mL electrolyte solution, which corresponds to 0.87 mg of PS. The influence of such low residual TOC contents on *FE*_plastic‑to‑CO2_ is marginal, leading to a decrease by up to ca. 1%: Our analysis of the TOC content does not lead to an overestimation of the mineralization efficiency.

### 2.12 GC Measurements and Calculation of Faradaic Efficiencies of H_2_ and O_2_ Production

H_2_ and O_2_ evolutions were quantified via GC, using a closed chamber under argon atmosphere, partially filled with electrolyte, as previously described.^38^ Before measurement, the electrolyte was degassed by evacuation of the chamber and subsequent purging with Ar. A Keithley Source Meter 2601 was used as potentiostat for chronoamperometric measurements, i.e. a fixed voltage was applied and the current *I*_exp_ was measured as a function of time. In the same time window, the gas composition inside the chamber was determined with an Inficon micro-GC fusion with the ability to detect molecular hydrogen, oxygen and nitrogen.

The total amount of gas inside the chamber *n*_tot_ was calculated using eq 8

$$n_{tot}=\frac{P\times V}{R\times T}$$ 8

where the gas volume in the chamber *V* = 0.000406 m^3^, the temperature *T* = 295.15 K, and the pressure inside the chamber *P* = 120000 Pa, giving the total amount of gas inside the chamber *n*_tot_ = 0.01985 ± 0.00071 mol. The measured molar fractions of H_2_ and O_2_, *c*_H_2__ and *c*_O_2__, were used to calculate the amount of evolved H_2_ and O_2_, *n*_*H*_2_,exp_ and *n*_O_2_,exp_ using eqs 9 and 10:

9 $$n_{H_{2},\exp}=n_{tot}\times c_{H_{2}}$$

10 $$n_{O_{2},\exp}=n_{tot}\times c_{O_{2}}$$

The expected amounts of hydrogen and oxygen *n*_H_2_,*q*_ and *n*_H_2_,*q*_ based on the charge were calculated using eq 11

$$n_{H_{2}{/O}_{2},q}=\frac{Q}{z\times F}$$ 11

where *Q* is the electric charge obtained by integrating over *I*_exp_, *z* represents the number of electrons used, e.g. *z* = 2 for H_2_ and *z* = 4 for O_2_, and *F* is the Faraday constant, e.g. *F* = 96485 C mol^–1^.

The system was calibrated by performing standard water splitting electrolysis at 2.1 V using two platinum wires immersed in 200 mL of 0.5 M H_2_SO_4_ for 5 h, assuming a 100% Faradaic efficiency under these standard conditions. The concentrations of H_2_ and O_2_ were measured every 30 min. After three different electrolysis experiments, the following calibration factors were experimentally defined: *cal*_*HER*_ = 0.994 ± 0.006 and *cal*_*OER*_ = 0.915 ± 0.006 for the HER and OER, respectively.

Faradaic efficiencies were determined using a Pt mesh as the cathode and a NATO-coated Ti as the anode. 80 mL of 0.75 M LiClO_4_ was used as the electrolyte solution, mixed with different types of plastic particles. During the GC experiments the electrolyte was stirred at 250 rpm, and a constant potential of 3 V was applied for 5 h. The hydrogen and oxygen concentrations inside the chamber were measured every 30 min. The experimental and expected amounts of H_2_ and O_2_ were calculated using eqs 8–11. The Faradaic efficiencies of the HER and the OER, FE_H_2__ and FE_O_2__, were obtained for a given electroreforming condition using eq 12 and 13:

12 $${FE}_{H_{2}}=\frac{n_{H_{2},\exp}}{{cal}_{HER}\times n_{H_{2},q}}$$

13 $${FE}_{O_{2}}=\frac{n_{O_{2},\exp}}{{cal}_{OER}\times n_{O_{2},q}}$$

### 2.13 Energy Efficiency (EE) of H_2_ Generation

The energy efficiency EE (in %) of the reforming process was calculated using eq 14:^39^

14 $$EE\;\left(\%\right)=\frac{\left(\frac{39\;Wh}{g}\right)\times \left(H_{2}\;rate\right)\times \left(\frac{2\;g}{mol}\right)}{E_{cell}\times I_{cell}}\times 100$$

where 39 Wh/g is the H_2_ specific heat of combustion, H_2_ rate is the rate of generation of hydrogen molecules in mol/s, *E*_cell_ (V) is the cell voltage, and *I*_cell_ (A) is the average cell current.

### 2.14 Energy Consumption and Estimated Electroreforming Costs

The energy consumed *E*_consumption_ (kWh/g) per reforming experiments was calculated with eq 15:

$$E_{\mathrm{consumption}}=\frac{Q_{\mathrm{reforming}}\times U}{3.6{\times 10}^{6}}\times \frac{1}{m_{\mathrm{plastic mineralized}}}$$ 15

where *U* is the applied potential in volts (V).

Electroreforming costs were estimated assuming energy costs of 0.02$/kWh, which is an optimistic but reasonable assumption.

## 3 Results and Discussion

The plastic particles used in this work are hydrophobic, derived from commercially available polystyrene (PS) and polypropylene (PP) objects, i.e. wastes, and have not been chemically treated before electroreforming (Figure 2). As such, they are good model systems for real-life plastic waste particles that could be prepared at the industrial scale or potentially found in polluted water. Electrochemical reforming of water-insoluble C-only plastic wastes is demonstrated in aqueous solution with FE_plastic‑to‑CO2_ up to 32% at plastic degradation efficiencies >80%, e.g. >80% of plastic converted into CO_2_. H_2_ production is demonstrated with a Faradaic efficiency FE_H2_ of up to 57%, and an energy efficiency EE of up to 30%. Compared to previous works, a significant improvement is reported: The lowest estimated energy consumption of our plastic reforming electrolyzer is ca. 0.10 kWh/g of plastic decomposed, which is >120 times lower than previously reported for the anodic mineralization of water-soluble plastic particles at BDD electrodes.^30^

**Figure 2:**
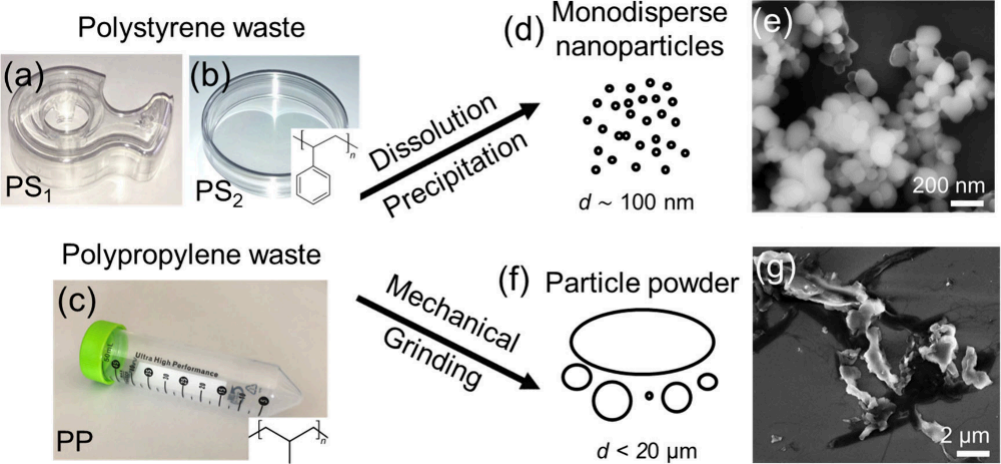
Plastic waste particles. (a–c) Photographs of the plastic wastes used: polystyrene (PS) waste obtained from tape casing (a), referred to as PS_1_, and Petri-dish (b), referred to as PS_2_, and polypropylene (PP) waste obtained from centrifuge tube (c). The plastic wastes were used to prepare: (d) monodisperse nanoparticle dispersions with a particle diameter *d* ∼ 100 nm via dissolution–precipitation and (f) plastic powders composed of highly polydisperse particles with sizes up to ca. 20 μm via mechanical grinding. (e, g) Typical SEM images of the resulting nanoparticles (e) and particle powders (g).

### 3.1 Electromineralization of PS Nanoparticles

Electrochemical reforming was performed in a two-electrode configuration, in a 0.75 M LiClO_4_ electrolyte solution containing plastic waste particles at a concentration of 0.46 g/L. The NATO anode – Pt cathode separation was kept constant at ca. 1 cm. The NATO anode was prepared by successive dip-coating and annealing steps, using a Ti grid support (more details in the Materials and Methods), as previously reported.^36^ The PS wastes (Figure 2) were either tape casings (3 M Inc.), referred to as PS_1_, or Petri dishes (VWR Inc.), referred to as PS_2_, and the PP wastes were obtained from centrifuge tubes (VWR Inc.). Raman spectroscopy confirms the purity of the polymer wastes used (see Figure S4 in the Supporting Information). The plastic particles were prepared from the waste precursors either via dissolution/precipitation, yielding nanoparticles (NPs) with an average diameter of ca. 110 nm (Figure 2d,e, and Figure S5 for particle size distributions), or via simple mechanical grinding (Figure 2f,g) producing a powder composed of heterogeneous particles with sizes up to 20 μm. To keep the process as simple as possible, no chemical treatment was used, but a small amount (<1% by weight) of the surfactant Triton-X was added to facilitate the dispersion of the nanoparticles prepared via dissolution/precipitation. However, even in the presence of a surfactant, most of the nanoparticles are present as large aggregates at the air–water interface (Figure 1a,b). No Triton-X was added to the particle powder prepared via mechanical grinding. Both types of plastic particle stock solutions were then mixed with the electrolyte solution to form a noncolloidally stable dispersion of plastic particles. Control experiments where the plastic stock solution was mixed with the LiClO_4_ electrolyte solution showed no measurable loss of plastic mass during a period of 5 days under stirring, which demonstrates the high stability of the plastic particles in the electrolyte without electrochemical input. FE_Plastic‑to‑CO2_ was estimated using a simple mineralization reaction and by weighing the remaining solid plastic isolated via filtration (see eqs 2–7 in Materials and Methods). The residual organic content present in the electrolyte solution was estimated from total organic carbon (TOC) measurements performed on the filtrates, and taken into account to calculate FE_plastic‑to‑CO2_ (eq 7).

Electroreforming of PS nanoparticles prepared via dissolution/precipitation at 6 V applied potential and under moderate stirring leads to the rapid formation of a white foamy precipitate on top of the water surface, shown in Figure 1c. We believe that foaming is initiated by the large amount of H_2_ and O_2_ gas bubbles that are generated at such high potentials. Even under strong stirring, most particles eventually separate out of the solution and incorporate irreversibly into the foam: at 6 V, most of the plastic particles are not in contact with the electrolyte solution, which significantly reduces the electroreforming efficiency and complicates the reliable quantification of the mineralization efficiency. We found that a more moderate potential, i.e. 4.5 V, together with vigorous stirring (i.e., 1400 rpm) forces the particles to come into contact with the electrode and the electrolyte, suppressing foaming. After ca. 2 h, the initially white plastic precipitate “swimming” at the electrolyte surface disappears: The plastic particles are solubilized during the early stages of electroreforming, most likely due to the oxidation of the plastic particle surface. This is supported by Raman spectroscopy of the remaining PS solid after electrolysis, filtration and drying, which shows the presence of oxygen-containing groups such as OH, COOH or COO^–^ (Figure S6). Although simple, we believe that this process is essential to efficiently electroreform hydrophobic C-only plastics.

At 4.5 V, almost all the PS_1_ particles are fully mineralized within 10 h, with a degradation efficiency of 93% and FE_PS1‑to‑CO2_ of ca. 14% (Figure 3a). FE_PS1‑to‑CO2_ significantly decreases during the reaction, going from ca. 45% after 1 h of electrolysis, to ca. 14% after 10 h of electrolysis, similarly to the decrease of FE_PS‑to‑CO2_ from 6% to 1% during electromineralization reported on BDD electrodes at 11 V.^30^ Such decrease in FE during electromineralization is common in water electrodecontamination systems and is often due to mass-transport limitations and incomplete mineralization in the early stages of the experiment. A controlled experiment performed at +4.5 V in the presence of a hydroxyl radical scavenger, methanol^36^ (concentration: 0.2 mol L^–1^), shows a clear decrease in degradation efficiency and FE_PS‑to‑CO2_, which go down to 32% and ca. 4.5% after 10 h. Methanol is known to efficiently scavenge HO^•^ radicals at the anode surface (*k*_HO^•^_ = 9.7 × 10^8^ M^–1^ s^–1^), which suggests that HO^•^ radical-mediated oxidation near the NATO surface plays an important role in the mineralization process.^36^ At the present, the exact degradation pathway is unknown, but the following chemical reaction sequence might occur as suggested by other groups:^30,40^ hydrogen abstraction induced by the HO^•^ radical, generating a carbon centered radical that can react with molecular oxygen to form a peroxyl radical. The peroxy radicals produce stable OH groups on the polymer chains. Further recombination and reaction transform the −OH groups into carboxylic acids, carbonyl groups, and other groups. After further reaction, the PS is eventually fully mineralized. This HO^•^-mediated oxidation is supported by the presence of OH, COOH and COO^–^ groups in the filtered and dried PS particles that remain after electrolysis (Figure S6).

**Figure 3:**
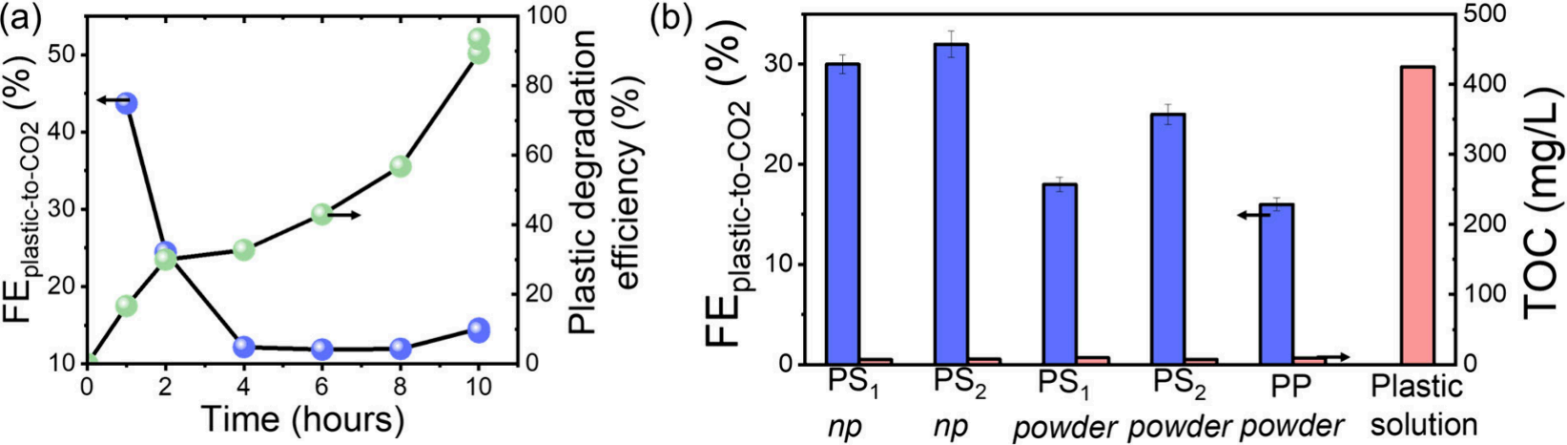
Plastic waste mineralization. (a) Electroreforming at 4.5 V of PS_1_ nanoparticles prepared via dissolution/precipitation. FE_PS1‑to‑CO2_ (left axis, blue circles) and plastic degradation efficiency (right axis, green circles), as a function of electroreforming duration. Two data points are provided for 10 h of electroreforming. (b) FE_plastic‑to‑CO2_ (blue bars, left axis) after 30 h electroreforming at 3 V of PS_1_, PS_2_ and PP particles. *np*: nanoparticle dispersions prepared via dissolution/precipitation. Powders: particle powders prepared via mechanical grinding. The corresponding residual TOC is shown for each experiment (red bars, right axis). The expected TOC originating from the stock solution containing 0.46g/L of plastic particle is shown as a comparison, labeled Plastic solution.

When performed at 3 V, electroreforming slows down, but becomes even more efficient: after 30 h, 81% degradation efficiency can be achieved with a FE_PS1‑to‑CO2_ of ca. 30%, which is 30 times higher than previously reported on BDD.^30^ This is consistent with the recent mineralization of commercially available water-soluble PS nanoparticles on BDD electrodes: A lower current density, which corresponds here to a lower applied voltage, resulted in a slower mineralization but a higher electrochemical efficiency.^31^ After extensive electroreforming, the filtered electrolyte typically shows a TOC of around 10 mg/L (Figure 3b), indicating that less than 3% of the initial plastic remains as soluble organic compounds. This suggests that the plastic particles have been almost completely mineralized into CO_2_, consistent with previous findings using BDD electrodes.^30^

Similar results were obtained with the second polystyrene waste precursor PS_2_, e.g. the Petri-dish, with a FE_PS2‑to‑CO2_ = 32% and a plastic degradation efficiency of ca. 85% observed after 30 h. This demonstrates that electroreforming efficiency does not strongly depend on the origin of the PS waste.

### 3.2 Electromineralization of PS and PP Powders

The industrial significance of our method is demonstrated through the electroreforming of plastic waste that was manually ground into a fine powder composed of highly heterogeneous particles with sizes up to ca. 20 μm. Importantly, no surfactant was added to the plastic powder solution, and sonication was not utilized. After 30 h of electroreforming at 3 V, these nonuniform PS_1,2_ particles are mineralized with high Faradaic efficiencies of FE_PS1‑to‑CO2_ ≈ 18% and FE_PS2‑to‑CO2_ ≈ 25% (Figure 3b), which are lower than what was observed for nanoparticles prepared via dissolution/precipitation, but are still remarkably high. The generality of the approach to other C-only polymers was investigated by mechanically grinding a PP centrifuge tube (VWR, Inc. see Figure 2c). Electroreforming at 3 V of the PP particles yields similar conversion efficiencies: after 30 h, FE_PP‑to‑CO2_ = 16% is achieved. These results demonstrate that electroreforming is not only limited to PS, but can also be used to efficiently degrade other types of C-only polymers, such as PP. All the particles investigated in this work are highly insoluble in water, forming large aggregates in the electrolyte solution. As a result, an accurate assessment of the particle size influence on plastic mineralization efficiency is difficult. However, since the FE_PP‑to‑CO2_ is lower for the particles prepared via grinding, we tentatively attribute this difference to the larger size of the pristine particles in these samples.

### 3.3 Energy Consumption of the Process

The energy consumption required to electroreform 1 g of plastic, and the corresponding energy costs that would be required to reform 1 ton of plastic particles are compared in Table 1, assuming energy costs of 0.02$/kWh (see SI Note I). The most efficient electroreforming was achieved at 3 V for PS_2_ nanoparticles, with FE_PS2‑to‑CO2_ = 32% and estimated energy costs of 1940$/ton, which is ca. 128 times less than what was previously reported on BDD at 11 V, requiring ca. 250 000$/ton of PS treated (see SI Note I and Table S1).^30^

**Table 1:** Electroreforming Efficiency, Energy Consumption, and Costs

Sample^a^	Duration (h)	Potential or current density	FE_Plastic‑to‑CO2_ (%)	Energy consumption (kWh/g)	Energy costs ($/ton)^b^
PS_1_, insoluble np	10	4.5 V	14	0.32	6480
PS_1_, insoluble np	30	3 V	30	0.10	2350
PS_1_, insoluble powder	30	3 V	18	0.18	3500
PS_2_, insoluble np	30	3 V	32	0.10	1940
PS_2_, insoluble powder	30	3 V	25	0.12	2460
PP, insoluble powder	30	3 V	16	0.22	4440
Not this work ref 30, PS, soluble mp on BDD	6	11 V	1	12.4	248000
Not this work ref 31, PS, soluble np on BDD	8	50 mA cm^–2^	n.a.	23.6	472297

a Definitions: np, nanoparticles prepared via dissolution–precipitation with an average diameter of 120 nm for PS1 and 105 nm for PS2; powder, plastic particles prepared via mechanical grinding; mp, water-soluble and commercially available PS microspheres used in ref 30, 150 nm water-soluble and commercially available PS nanoparticles used in ref 31.

b Calculated using energy costs of 0.02$/kWh.

### 3.4 Electroreforming Plastic Wastes and Hydrogen Production Efficiency

To assess the economic viability of electroreforming, the amount of molecular hydrogen and oxygen generated was quantified via gas chromatography (GC, measurement details in the SI), presented in Figure 4. Without plastic particles, low Faradaic efficiencies are achieved on Pt and NATO electrodes with FE_H2_ = 37% and FE_O2_ = 34% (Figure 4a), with a H_2_/O_2_ ratio around 2.2. We attribute these low FEs to the generation of reactive water oxidation products, such as O_2_, H_2_O_2_, O_3_ and HO^•^ radicals at the NATO anode,^36,37^ and their preferential crossover reduction at the Pt cathode due to their high standard electrochemical potentials. The large deviation from 100% may also result from ohmic losses through the Ti-NATO interface, and oxidative dissolution of the NATO anode, known to beneficially participate in the generation of reactive oxygen species.^37^ Energy dispersive X-ray spectroscopy (EDS), X-ray diffraction (XRD) and Raman spectroscopy measurements show that extensive electrolysis, i.e. > 100 h at 3 V, leads to a decrease in the relative atomic content of Ni and Sb (relative to Ti, Figure S1), and intensities of both the XRD peaks (Figure S2), and Raman bands of pristine rutile SnO_2_ (Figure S3). Additionally, we measured an average weight loss of the electrode of ca. 22 mg/1000C at 3 V in 0.75 M LiClO_4_. Assuming that the corrosion of the NATO/Ti electrode occurs as a two-electron oxidation to generate H_2_O_2_ (eq 16)^37^

**Figure 4:**
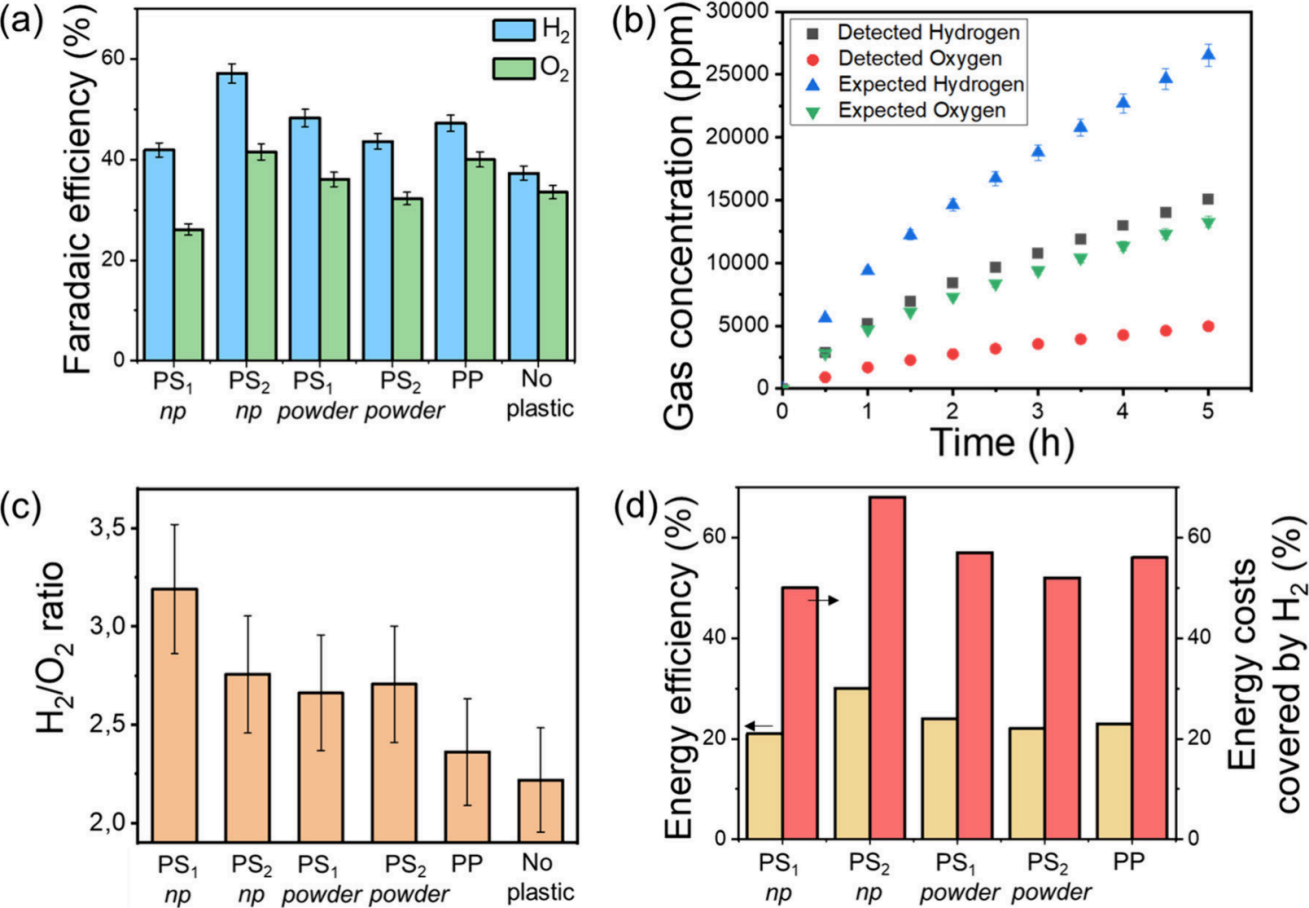
H_2_ generation during plastic electroreforming. (a) FE_H2_ (in blue) and FE_O2_ (in green) measured via GC for different plastic particles. The control electrolysis experiment performed in pure 0.75 M LiClO_4_ with a Pt cathode and NATO anode is labeled “No plastic”. (b) Detected H_2_ (black square) and O_2_ (red circles) concentrations in the chamber as a function of time during the electroreforming of PS_2_ nanoparticles. The expected gas concentration corresponding to 100% Faradaic efficiency is provided as a guide for both H_2_ (blue triangles) and O_2_ (green inverted triangles). (c) Measured H_2_/O_2_ ratio. (d) Energy efficiency of H_2_ generation (light orange bars, left axis) and estimated percentage of the energy costs that could be recovered by the sale of the H_2_ produced during the electroreforming experiment (red bars, right axis).

16 $${\mathrm{SnO}}_{2}+2H+\to {\mathrm{Sn}}^{4+}+H_{2}O_{2}+2e^{-}$$

we estimate that ca. 2.8% of the anodic current is used to corrode the NATO coating. Taken together, these results suggest that our NATO anodes suffer from stability issues under our experimental conditions, which might contribute to the low Faradaic efficiencies of H_2_ and O_2_ evolution.

Upon addition of plastic particles, FE_H2_ (Figure 4a, b) and the H_2_/O_2_ ratio (Figure 4c) increase for all samples, reaching a maximum of 57% and 3.2 for the nanoparticles PS_2_ and PS_1_, respectively. This indicates that oxidation species at the NATO anode effectively react with plastic particles, reducing crossover reactions and enhancing hydrogen generation efficiency. Additionally, the 30–40% FE_O2_ measured in the presence of plastic particles is compatible with the high values of FE_plastic‑to‑CO2_ obtained in this work, e.g. in the 14–30% range. This indicates that our simple description of the anodic mineralization process (see eqs 2–5) is consistent with the amount of anodically produced O_2_ measured via GC.

A maximum energy efficiency (EE, in %, see experimental section for more details) of H_2_ generation was obtained during the electroreforming of PS_2_ nanoparticles, with EE = 30% (Figure 4d, light orange bars). Because H_2_ has a high market value of ca. 1900$/ton, it is possible to recoup some of the energy costs by selling the H_2_ produced (Figure 4d, red bars). We estimate that up to 68% of the energy costs consumed during the electroreforming of PS_2_ nanoparticles could be recovered by selling the H_2_ produced at the cathode, which is not negligeable. An even greater portion of the energy costs could be regained by carrying out reforming at even lower applied potentials to increase EE_H2_ and FE_plastic‑to‑CO2_. Additionally, a two-compartment electrolyzer equipped with a cation conducting membrane could be used to separate the cathodic and anodic products: This would prevent crossover reactions, most likely increasing H_2_ generation efficiency, while minimizing both the risks associated with mixing of O_2_ with H_2_, and the costs necessary for gas separation.

### 3.5 Estimated CO_2_ Emissions

We estimated the total CO_2_ emissions required to produce 1 kg of H_2_ via plastic electroreforming, taking into account the energy input, and direct emissions originating from plastic mineralization. We find that it ranges from ca. 1.65 to 13.02 kg_CO2eq_/kg_H2_ depending on the electricity origin (nuclear, solar, hydro or wind) and plastic concentration (see note S2 and Table S2). The contribution of direct CO_2_ emissions, i.e. CO_2_ released during plastic mineralization, is insignificant under our current conditions, i.e. 0.014 kg_CO2eq_/kg_H2_. The electron balance supports a maximum of 0.723 kg of PS mineralized for each kg of H_2_ produced, corresponding to a plastic concentration of ca. 80g/L (see Note S2). This provides an upper boundary of the direct CO_2_ emission of the process, i.e. 2.44 kg_CO2eq_/kg_H2._ Overall, these numbers compare remarkably well with conventional plastic waste gasification to produce H_2_, which emit ca. 12 kg_CO2eq_/kg_H2_.^41^ If scalable, electroreforming could serve as a much softer method for producing H_2_ while simultaneously mineralizing plastic wastes. Thanks to the portability and tunability of electrochemical systems, our approach should be well-suited for integration with downstream carbon capture and storage to minimize CO_2_ emissions. Alternatively, plastic electroreforming under alkaline conditions could offer a direct method to electroreform plastics while directly capturing and storing CO_2_ *in situ*. This is currently under investigation in our laboratories.

## 4 Conclusion

To conclude, we report here the efficient electroreforming of chemically resilient and hydrophobic C-only plastic wastes of different origins, particle shapes and size distributions. FE_plastic‑to‑CO2_ as high as 32% are reported at low potentials, i.e. 3 V, with a significant improvement in energy consumption by factors ranging between ca. 56 to ca. 128 compared to previous mineralization results of water-soluble synthetic PS microspheres.^30^ The polymer particles are expected to be mineralized by HO^•^ radicals. The compatibility of electroreforming real-life plastic wastes prepared via grinding, which is simple and cost-effective, paves the way for potential industrial applications. If scaled-up to the treatment of large amounts of plastic wastes, electroreforming would require, for now, energy costs of ca. 2000–5000$/ton of plastic wastes treated. While still too high, this is significantly lower than ca. 250 000 $/ton of plastic waste that was previously reported (see SI Note I).^30^ When extrapolating CO_2_ emissions from water electrolyzers (see Note S2 and Table S2) and taking into account direct CO_2_ emissions due to plastic mineralization, our plastic electroreforming conditions compare very well with more mature waste-to-H_2_ technologies such as gasification. An estimated CO_2_ emission as low as 1.65 kg_CO2eq_/kg_H2_ is estimated if nuclear energy is used, which is excellent considering the fact that plastics are degraded in the process of producing H_2_. However, before scaling up, several key challenges must be addressed. First, the mineralization efficiency needs further improvement, likely achievable through reactor and electrode engineering. Given the solid nature of plastic particles, optimizing mass transport could provide significant efficiency improvement. This could be done using flow-through cell architectures and anodes with hierarchical porosity to facilitate access to the electrode surface of both large aggregates present in the stock solution, and smaller particles formed during the reaction. Additionally, finding cost-effective, and high-performance anodes that are stable over thousands of hours will be essential.

In its current state, electroreforming could recover ca. 70% of the energy input costs by selling the hydrogen produced. The efficiency improvements reported here are substantial compared to previous findings and are anticipated to encourage other research groups to explore the use of electrochemistry for reforming and upcycling resilient C-only plastic solid wastes. Our work expands the scope of electrochemical polymer conversion, which has previously been limited to oxygen-containing polymers due to their lower chemical stability and greater electrochemical reactivity. As such, this study might inspire other groups to use electrochemistry to treat C-only plastic wastes.

## Supplementary Material



## Notes

Energy consumption and cost estimates, life cycle assessments. EDS data, XRD diffractograms, Raman spectra, and PS nanoparticle size distributions (PDF)

## Abbreviations

BDD: boron-doped diamond
EE: energy efficiency
FE: Faradaic efficiency
FE_H2_: Faradaic efficiency of H_2_ generation
FE_O2_: Faradaic efficiency of O_2_ generation
FE_plastic‑to‑CO2_: Faradaic efficiency of the plastic mineralization (conversion into CO_2_)
NATO: nickel antimony tin oxide
PET: polyethylene terephthalate
PP: polypropylene
PS: polystyrene
TOC: total organic carbon
