# Supplementary material for: Efficient Electrochemical Reforming of Water-Insoluble C‑Only Plastic Wastes
Source: ACS Sustain Chem Eng. 2025 May 27;13(22):8289–97. doi: 10.1021/acssuschemeng.5c00907 (PMC12153041; doi:10.1021/acssuschemeng.5c00907)
Supplement: Supplementary file 1 [file sc5c00907_si_001.pdf]

Supporting Information

Efficient Electrochemical Reforming of Water-Insoluble C-Only Plastic Wastes

Tayebeh Esmaeili, Julian Hörndl, Simone Pokrant, Theresa Bartschmid, Amin Farhadi, and Gilles R. Bourret*

Department of Chemistry and Physics of Materials, University of Salzburg, Jakob Haringerstraße 2a, A-5020 Salzburg, Austria

*E-mail: gilles.bourret@plus.ac.at

KEYWORDS: reforming, hydrogen, plastic waste, polystyrene, polypropylene, electrocatalysis

Table of content

Note S1. Estimation of the energy consumption and costs for the electromineralization of polystyrene microparticles and nanoparticles based on the previous work from Kiendrebeogo et al.1, and Perez-Lopez et al.2, respectively. S2

Table S1. Energy consumption reported by reference 2. .... S2

Note S2. Life cycle assessment of technologies producing hydrogen from plastic wastes. S3

Table S2. Estimated CO_2 emissions, expressed as kg_{CO2eq}/kg_{H2} S5

Figure S1. Normalized EDX atomic % ratios of Sn/Ti, Ni/Ti and Sb/Ti S6

Figure S2. XRD diffractograms S6

Figure S3. Raman spectra of NATO/Ti electrodes S7

Figure S4. Raman spectra of the solid plastic waste precursors used. S8

Figure S5. Size distribution of the polystyrene PS1 (a) and PS2 (b) nanoparticles S9

Figure S6. Raman spectra of the pristine plastic S9

References. S10

Note S1. Estimation of the energy consumption and costs for the electromineralization of polystyrene microparticles and nanoparticles based on the previous work from Kiendrebeogo et al.1, and Perez-Lopez et al.2, respectively.

Kiendrebeogo *et al.* report a consumption of 1120 kWh/m^3 containing 100mg/L of PS microparticles at a ca. 90% degradation efficiency (i.e. 90% of PS degraded).[1] This corresponds to 1120 kWh/90g of PS, or 12.4kWh/g of PS. If upscaled, the process would require prohibitive energy costs of 248 000$/ton, assuming electricity costs of 0.02$/kWh. Such a low energy price is an optimistic but reasonable assumption based on the use of renewable energy conversion systems: In the US, the projected price for photovoltaic-based electricity as low as 0.03$/kWh is expected in the near future, with a similar trend for wind energy, with wind electricity price already being ~ $0.02/kWh.[3, 4]

Perez-Lopez et al. report a consumption between 37.16 and 1317.78 kWh/m^3 containing 20mg/L of PS nanoparticles at various degrees of degradation efficiencies and mineralizaton Faradaic efficiencies.[2] We summarize in **Table S1** here the results reported in Reference [2].

Table S1. Energy consumption reported by reference 2.

Current density (mA.cm-^{2})	Electrolysis duration	Degradation efficiency	Energy consumption (kWh/m^3)	Mass of PS degraded per m^3	Energy consumption (kWh/g)	Energy costs ($/ton)[b]
10	7h	50%	37.16	10g	3.7	74 320
50	2.80h	50%	151.47	10g	15.1	302 940
50	8h	91%	429.79	18.2g	23.6	472 297
100	1.30h	50%	197.49	10g	19.7	394 980
100	8h	97%	1317.78	19.4g	67.9	1 358 536

In the main text, we compare our results in **Table 1** with previous results corresponding to conditions where the PS was significantly degraded, i.e. > 80% degradation efficiency:

- Ref. [1], for which we estimated energy consumption estimated at 12.4 kWh/g of PS
- Ref. [2], for which we selected the lowest reported energy consumption, i.e. 50 mA.cm^{-2} during 8 hours.

The energy consumption we report is much lower than what was reported in both of these previous results (compare Table S1 and Table 1 in the main text). Even the most energy efficient electrolysis condition reported in ref. [2], achieved after 7 hours at 10mA.cm^{-2} on BDD with a degradation efficiency of only 50%, i.e. incomplete mineralization, results in an estimated energy consumption of 3.7 kWh/g, which is still 37 times higher than our best results.

Note S2. Life cycle assessment of technologies producing hydrogen from plastic wastes.

Here, we compare the kg_{CO2eq}/kg_{H2} generated by plastic electroreforming (this work), water electrolysis and waste polymer gasification. These numbers are only provided as estimates to qualitatively compare our process with other technologies and should not be taken as absolute numbers. The results are summarized in Table S2.

Emissions from water electrolysis to produce H_2

The following estimations were extracted from reference 5 for water electrolysis (proton exchange membranes) powered *via* renewable electricity (wind, hydroelectric, and solar), which ranges from 2.05 to 4.96 kg_{CO2eq}/kg_{H2}. 0.77 kg_{CO2eq}/kg_{H2} was used for water electrolysis using nuclear energy according to reference 5.

Emissions from plastic electroreforming to produce H_2

Contribution of energy input: To estimate the amount of CO_2 generated by electroreforming, we extrapolated these numbers based on the energy efficiency of H_2 production by our process, i.e. ca. 30%, and assuming an energy efficiency of 64% for water electrolysis:[6] For example, plastic electroforming powered by nuclear energy would generate $0.77 \times \frac{64}{32} = 1.64$ kg_{CO2eq}/kg_{H2}. For the sake of simplicity, we assume here that our process could be directly used with state-of-the-art flow cells equipped with proton exchange membranes.

Contribution of direct emission due to the complete plastic mineralization into CO_2: Assuming a minimum of 9 liters of H_2O to produce 1kg of H_2, and reforming with a plastic concentration of 0.46g/L, we would expect the CO_2 emission arising from the mineralization of the plastic to be insignificant, e.g. $0.46\ [\frac{g}{L}] \times 9[L] = 4.14$ g of polystyrene used, corresponding to ca. 0.014 kg of CO_2 emitted per kg of H_2 produced. This assumes that the complete mineralization of 1 kg of polystyrene generates ca. 3.38 kg of CO_2, calculated as the ratio $8 \times M_w(CO_2)/ M_w(C_8H_8)$,

where $M_w(CO_2)$ is the molecular weight of CO_2 and $M_w(C_8H_8)$ the molecular weight of the PS repeating unit C_8H_8.

Estimation of the upper boundary of direct emission: With FE_{H2} and $FE_{PS\text{-}to\text{-}CO2}$ of 57% and 32%, respectively, we estimate that the electrons consumed to generate 1kg of H_2 would be able to mineralize ca. 723 g of PS, which corresponds to the maximum concentration of plastic that could be mineralized without wasting energy. This is quite high, i.e. ca. 80g PS/L, but provides an upper boundary to estimate the maximum direct emission, i.e. $0.723 \times 3.38 = 2.44$ kg_{CO2eq}/kg_{H2}.

Estimated cumulated CO_2 emission: The cumulated CO_2 emission of electroreforming PS was estimated by adding the emission originating from the energy input, which depends on the electricity source, and the direct emissions, which are set by the plastic concentration. As such, the maximum cumulated emission would be at most $X + 2.44$ kg_{CO2eq}/kg_{H2}, where X is the emission related to the energy input. To provide a fair comparison with gasification technologies that convert large amounts of plastic wastes, we provide a range of cumulated plastic electroreforming emissions going from X+0.014 to X+2.44 kg_{CO2eq}/kg_{H2}.

Emissions from plastic waste gasification to produce H_2

The emitted kg_{CO2eq}/kg_{H2} by waste polymer gasification was estimated using reference 5 which compares the life cycle assessments of several H_2 production technologies. Salah et al. report 9.75 kg_{CO2eq}/kg_{H2} for a mixture of 63.4 wt % PE, 30 wt % PP, and 6.6% wt % PS, taking into account 2.42 kg_{CO2eq}/kg_{H2} of "CO_2 savings" which originates from the fact that these polymers will not release CO_2 via incineration or landfill. Thus, polymer to H_2 gasification is estimated at ca. 12.17 kg_{CO2eq}/kg_{H2} by reference 5.

Table S2. Estimated CO_2 emissions, expressed as kg_{CO2eq}/kg_{H2}

Technology	Plastic	Electricity generated kg_{CO2eq}/kg_{H2}	Emission generated $kg_{CO2eq}/kg_{plastic}$	Cumulated kg_{CO2eq}/kg_{H2}
Water electrolysis (renewable energy)	n.a.	2.05-4.96	n.a.	n.a.
Water electrolysis (nuclear energy)	n.a.	0.77	n.a.	n.a.
This work: Plastic electroreforming – renewable energy	PS	4.37 – 10.58	0.014 – 2.44	4.38 - 13.02
This work: Plastic electroreforming – nuclear energy	PS	1.64	0.014 – 2.44	1.65 - 4.08
Waste polymer gasification from Ref. [5]	Mixture PE, PS, PP	9.75	2.42	12.17

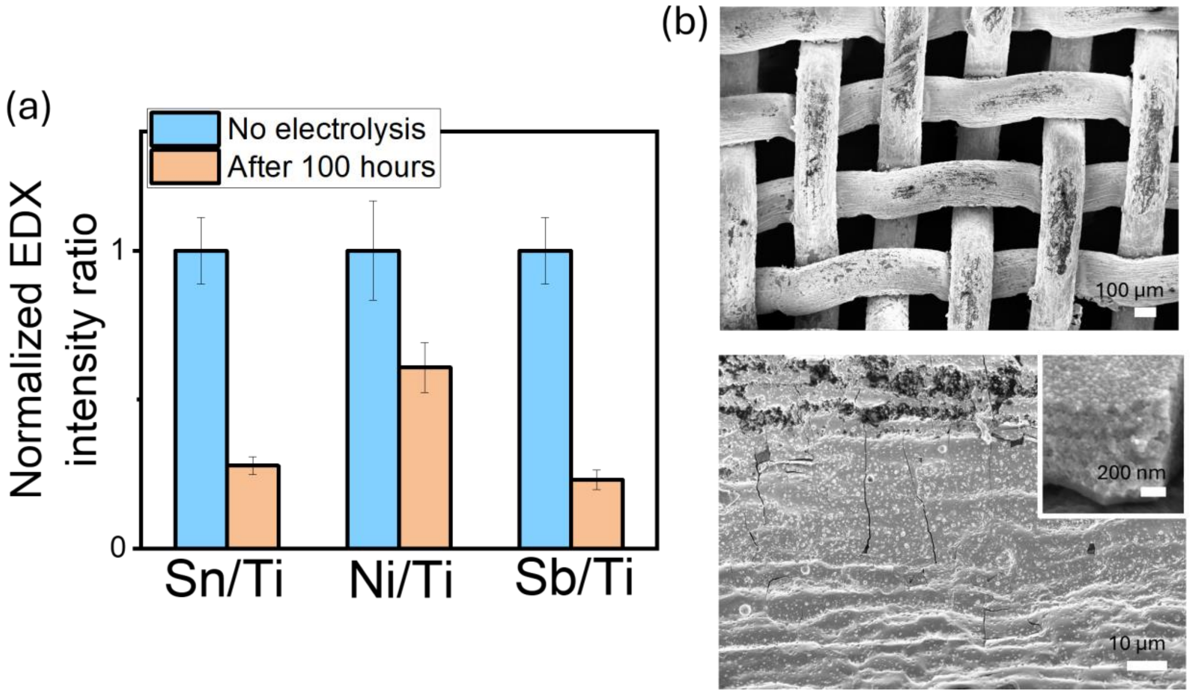

Figure S1. (a) Normalized EDX atomic % ratios of Sn/Ti, Ni/Ti and Sb/Ti **measured before and after extensive electrolysis.** The results are relative to the Ti signal because the roughness and porous geometry can affect the X-Ray emission yield and collection by the EDX detector, mostly due to shadowing effects. The error bars represent the standard error for each ratio. (b) SEM images of the pristine NATO/Ti mesh electrode.

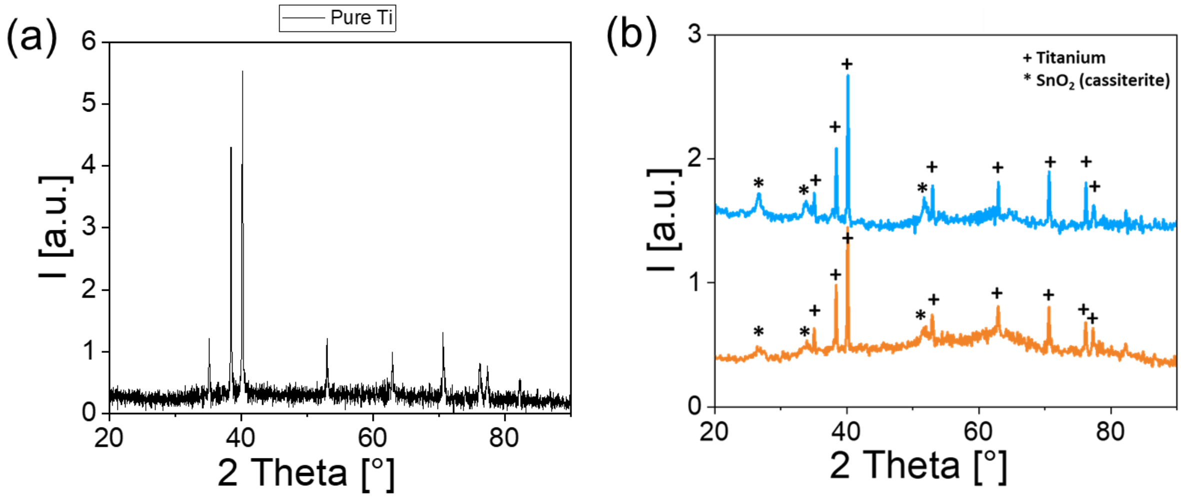

Figure S2. XRD diffractograms of (a) the pristine Ti mesh (i.e. no SnO_2, raw data), and (b) of the NATO/Ti mesh (smoothed data), before (light blue line) and after electrolysis (orange line). The position of the expected peaks for Ti and SnO_2 are shown with the plus sign and the star sign, respectively. The diffractograms were offset in (b) for clarity. A decrease in the SnO_2 peak intensities can be seen after electrolysis in (b).

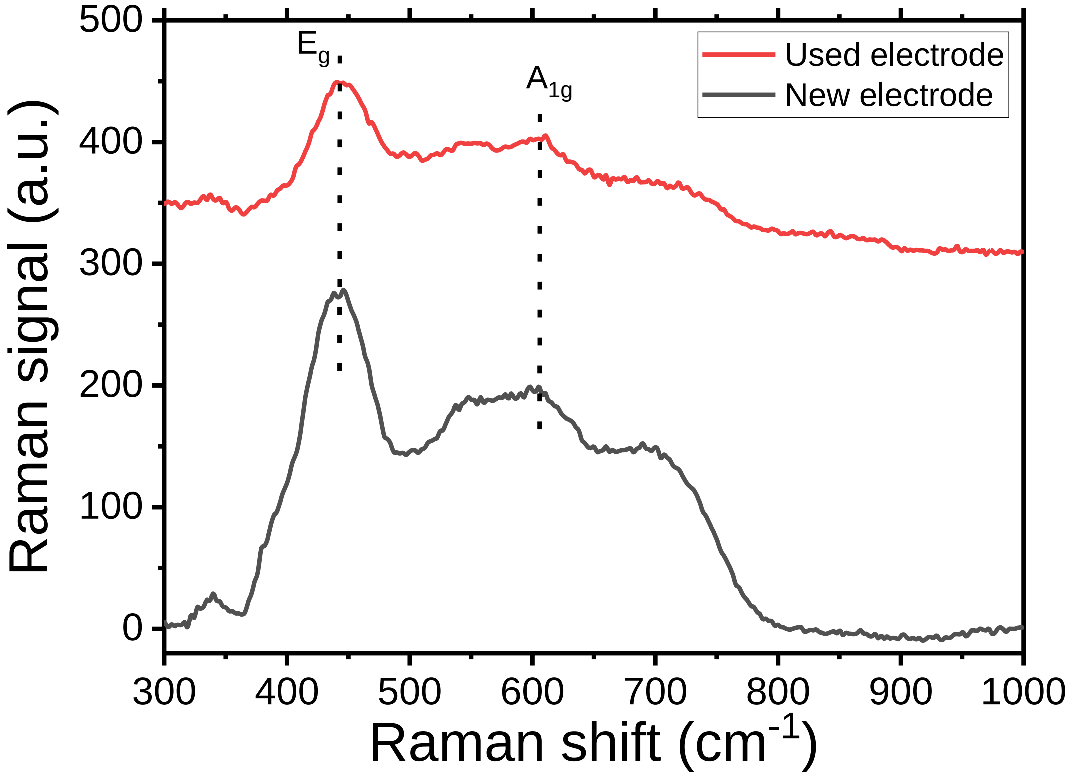

Figure S3. Raman spectra of a NATO/Ti electrode before (a) and after (b) electrolysis. The characteristic E_g and A_{1g} bands, expected to be present at 439 cm^{-1} and 610 cm^{-1}, respectively,[7] are identified in both cases at ca. 442 cm^{-1} and 606 cm^{-1} for the pristine NATO (a) and at ca. 444 cm^{-1} and 610 cm^{-1} after electrolysis (b). The decrease in the band intensity suggests that the NATO electrode integrity is affected by electroreforming.

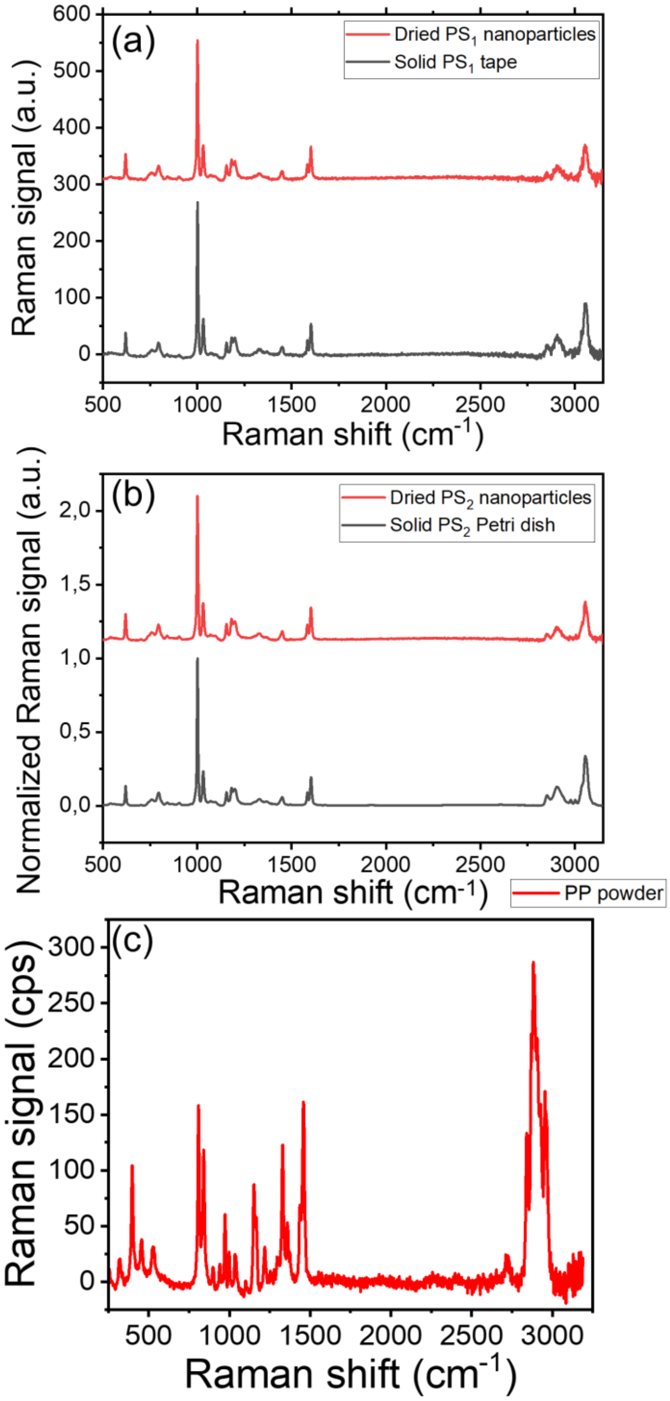

Figure S4. Raman spectra of the solid plastic waste precursors used. (a, b) PS_1 (top) and PS_2 (bottom) waste before any treatment (black curves) and, of the nanoparticles prepared after dissolution in dichloromethane, precipitation in alcohol solution, and vacuum drying (red curves). All spectra show identical Raman peak positions and intensities, which can all be assigned to pure polystyrene. (c) As prepared polypropylene (PP) particle powder after mechanical grinding. The spectrum matches the typical Raman spectrum of pure polypropylene.

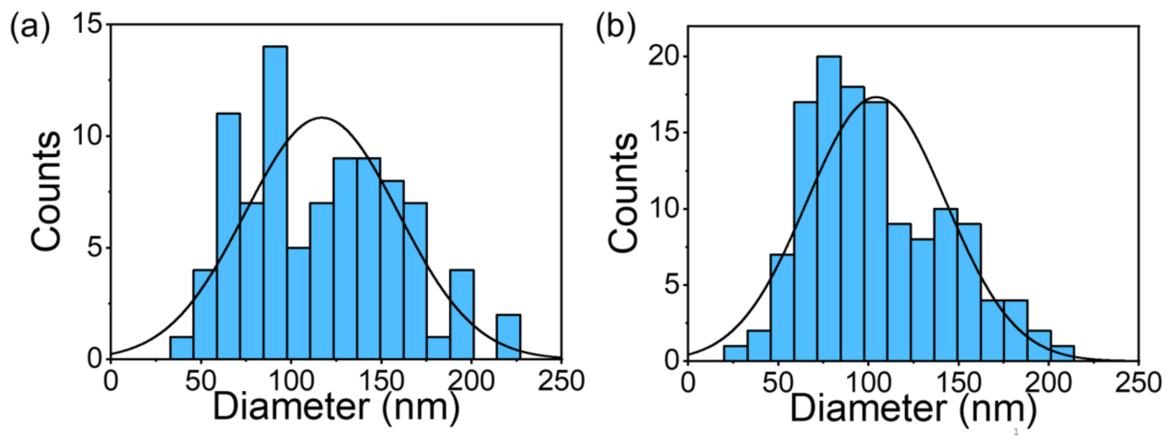

Figure S5. Size distribution of the polystyrene PS1 (a) and PS2 (b) nanoparticles prepared via dissolution/precipitation, with an average diameter of 120 ± 40 nm, and 105 ± 40 nm, respectively.

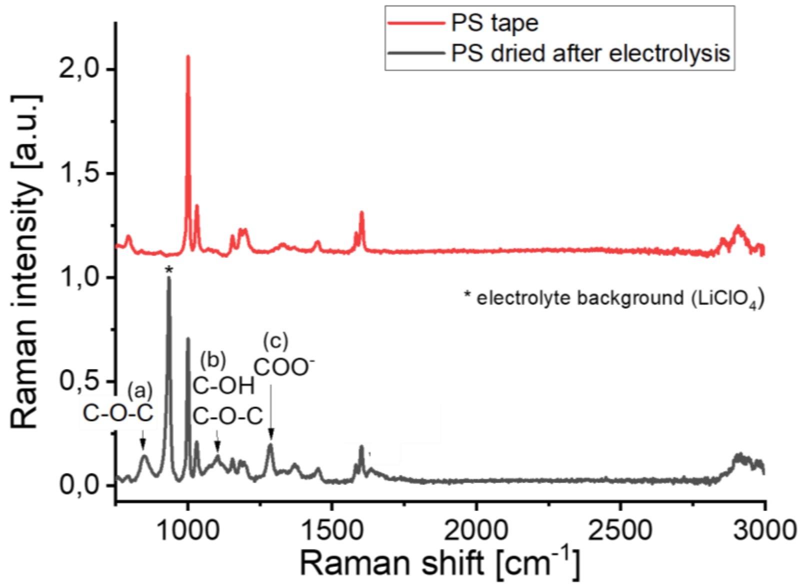

Figure S6. Raman spectra of the pristine plastic (in red) and of the PS particles after electrolysis, filtration and drying (in black). Peak (a) at ca. 850 cm^{-1} is tentatively assigned to $\delta(-C-O-C-)$ from ether groups; Peak (b) at 1104 cm^{-1} is tentatively assigned to $v\,(-C-O)$ from alcohol groups or ether groups; Peak (c) at 1285 cm^{-1} is tentatively assigned to $v\,(-C-OH)$ from alcohols, or symmetric $v\,(-COO^{-})$ from carboxylate groups.

References.

(1) Kiendrebeogo, M.; Karimi Estahbanati, M. R.; Khosravanipour Mostafazadeh, A.; Drogui, P.; Tyagi, R. D. Treatment of microplastics in water by anodic oxidation: A case study for polystyrene. *Environmental Pollution* **2021**, *269*, 116168. DOI: https://doi.org/10.1016/j.envpol.2020.116168.

(2) Pérez-López, A.; Domínguez, C. M.; Santos, A.; Cotillas, S. Removal of polystyrene nanoplastics from urban treated wastewater by electrochemical oxidation. *Separation and Purification Technology* **2025**, *363*, 132139. DOI: https://doi.org/10.1016/j.seppur.2025.132139.

(3) Wiser, R. B., M. Wind Technologies Market Report (US Department of Energy, 2016). **2016**. DOI: https://www.energy.gov/sites/default/files/2017/10/f37/2016_Wind_Technologies_Market_Report_101317.pdf.

(4) Shin, H.; Hansen, K. U.; Jiao, F. Techno-economic assessment of low-temperature carbon dioxide electrolysis. *Nature Sustainability* **2021**, *4* (10), 911-919. DOI: 10.1038/s41893-021-00739-x.

(5) Salah, C.; Cobo, S.; Pérez-Ramírez, J.; Guillén-Gosálbez, G. Environmental Sustainability Assessment of Hydrogen from Waste Polymers. *ACS Sustainable Chemistry & Engineering* **2023**, *11* (8), 3238-3247. DOI: 10.1021/acssuschemeng.2c05729.

(6) Nguyen, T.; Abdin, Z.; Holm, T.; Mérida, W. Grid-connected hydrogen production via large-scale water electrolysis. *Energy Conversion and Management* **2019**, *200*, 112108. DOI: https://doi.org/10.1016/j.enconman.2019.112108.

(7) Ding, L.; Li, W.; Xue, M.; Peng, X.; Shi, H.; Liu, J.; Wang, X.; Jiang, C.; Xue, Y.; Wang, S.; et al. Simultaneous ozone and hydrogen peroxide electrosynthesis via defect modulation in Ni, Sb-doped SnO2 electrocatalysts. *AIChE Journal* **2024**, *70* (3), e18314. DOI: https://doi.org/10.1002/aic.18314.
